# The Standardization and Comparability Gap in Evaluating Antibacterial Surfaces for Load-Bearing Orthopedic Implants: A Critical Review

**DOI:** 10.3390/bioengineering13090999

**Published:** 2026-08-27

**Authors:** Il-Hoon Kwak, Hyunsuk Choi

**Affiliations:** 1 Department of Orthopedic Surgery, Kwak Hospital, Daegu 41919, Republic of Korea; 2 Department of Dentistry and Prosthodontics, Daegu Catholic University School of Medicine, Daegu 42472, Republic of Korea

**Keywords:** periprosthetic joint infection, antibacterial coating, titanium implant, surface modification, evaluation methodology, standardization, osseointegration, biofilm

## Abstract

Periprosthetic joint infection is among the most serious complications of joint replacement, and the implant surface, where infection begins, is a rational prevention target. Antibacterial titanium surfaces act by releasing an agent, by contact killing, or by resisting adhesion. Yet the field cannot readily tell which method performs best, because efficacy is evaluated with methods that differ at every level and are rarely comparable: the same surface can read as highly effective under one assay and inactive under another, so the test rather than the material decides the verdict. This review asks where comparability is lost and what would restore it, for permanent load-bearing arthroplasty implants. We dissect the gap across in vitro assays, durability testing, surface characterization, in vivo models, clinical infection definitions, and regulatory expectations and ask whether the antibacterial–osseointegration trade-off is assessed coherently. We then propose a ten-item, stage-stratified minimum-reporting checklist and test it retrospectively on thirteen open-access studies. No study reported every item required at its stage; none related antibacterial dose to a stated cytotoxicity threshold; and studies fell short at different tiers, so their reported efficacies are not commensurable. The checklist is offered as a testable starting point for multi-stakeholder consensus, not a finished standard.

## 1. Introduction

Total joint arthroplasty is among the most successful procedures in modern surgery, but its worst complication—periprosthetic joint infection (PJI)—is disproportionately costly in morbidity, revision surgery, and health-system burden. The scale is not trivial, and it is growing. In the United States, the combined annual hospital cost of hip and knee PJI has been projected to reach roughly USD 1.85 billion by 2030, driven less by any rise in the per-case infection rate than by the expanding volume of primary arthroplasty [1]. Total joint arthroplasty is projected to exceed 1.9 million procedures a year by 2030 and five million by 2040 [2]. Because the infection rate per procedure has stayed broadly constant despite improvements in surgical technique [1], we argue that prevention increasingly depends on the implant itself rather than on the operative environment alone.

Infection of an implant begins at its surface. Circulating or locally introduced bacteria adhere to the implant, and once a biofilm has matured, it shields the organisms from both host immunity and systemic antibiotics, so that eradication usually requires revision surgery rather than antibiotics alone. The implant–tissue interface is especially vulnerable because it is a locally immunocompromised niche in which host cells and bacteria effectively compete for the surface—the “race for the surface” [3]—and a bacterial victory is difficult to reverse once biofilm is established. This sequence makes the implant surface itself a logical line of defense, and it explains why surface engineering has become one of the most active areas in orthopedic biomaterials.

The response has been a proliferation of antibacterial surface strategies. Titanium can be textured at the nanoscale to rupture bacterial membranes, loaded with antibiotics that elute over days to weeks, coated with silver or other metal ions, engineered to release osteogenic and antibacterial agents simultaneously, or modified in the plasma state to alter its chemistry and topography at once. Individually, many of these approaches report impressive reductions in bacterial adhesion, viability, or biofilm mass.

The difficulty is that these reports cannot be placed on a common scale. The methods used to demonstrate antibacterial efficacy differ in the bacterial strain and inoculum, in whether killing, adhesion, or biofilm is measured, in incubation time and quantification metric, and in whether any mechanical, corrosion, or in vivo testing accompanies the antibacterial claim. None of this is merely cosmetic. When identical surfaces are put through different standardized protocols, the measured efficacy can change enough that a material appears effective or ineffective according to the assay chosen [4], particularly when the assay does not match the surface’s mechanism of action [5].

Evaluation methodology has in fact received serious attention, but in fragments. Reviews of in vitro test-method adequacy [6,7], an assay-classification analysis within a strontium/silver systematic review [8], and a clinical-definition critique inside a network meta-analysis [9] each interrogate one tier of the evidence pipeline. What has not been done is connecting these tiers (in vitro assay, mechanical and corrosion durability, surface characterization, in vivo model, and clinical definition) into a single account of where comparability leaks, coupled to a cross-tier reporting proposal for antibacterial implant surfaces. That integration is the purpose of this review. Concretely, we set out to do three things: to establish where comparability is lost along the evaluation pipeline; to propose the minimum set of items whose reporting would make cross-study comparison possible; and to test whether that proposal actually separates comparable from non-comparable work when applied to published studies. Section 2, Section 3, Section 4, Section 5 and Section 6 follow the pipeline from the surface landscape to regulatory expectations, and Section 7, Section 8 and Section 9 develop, test, and situate the proposal. We are concerned less with the inventory of coatings than with the question of whether the evidence for them can be trusted and compared: a question that sits squarely within the “…and Evaluation” emphasis of this Special Issue. Our vantage point is deliberately that of implant surface science and evaluation methodology, transferable across implant fields, rather than the clinical management of infection, which lies outside our scope and expertise. We map the landscape only enough to organize the discussion, then concentrate on where and why comparability breaks down, and we close with a constructive proposal.

As a narrative critical review, this article does not attempt an exhaustive systematic search; relevant literature was identified primarily through PubMed/MEDLINE together with hand-searching of the reference lists of key reviews, and sources were selected by relevance to evaluation methodology rather than by systematic capture. Reporting follows the quality domains for non-systematic reviews described by the Scale for the Assessment of Narrative Review Articles (SANRA): importance and aims of the review, literature search, referencing, and presentation of evidence level [10]. The intentional consequence is that individual primary studies are cited illustratively to demonstrate a methodological point and are not offered as pooled or exhaustive evidence.

## 2. The Antibacterial Surface Landscape

**Figure 1:**
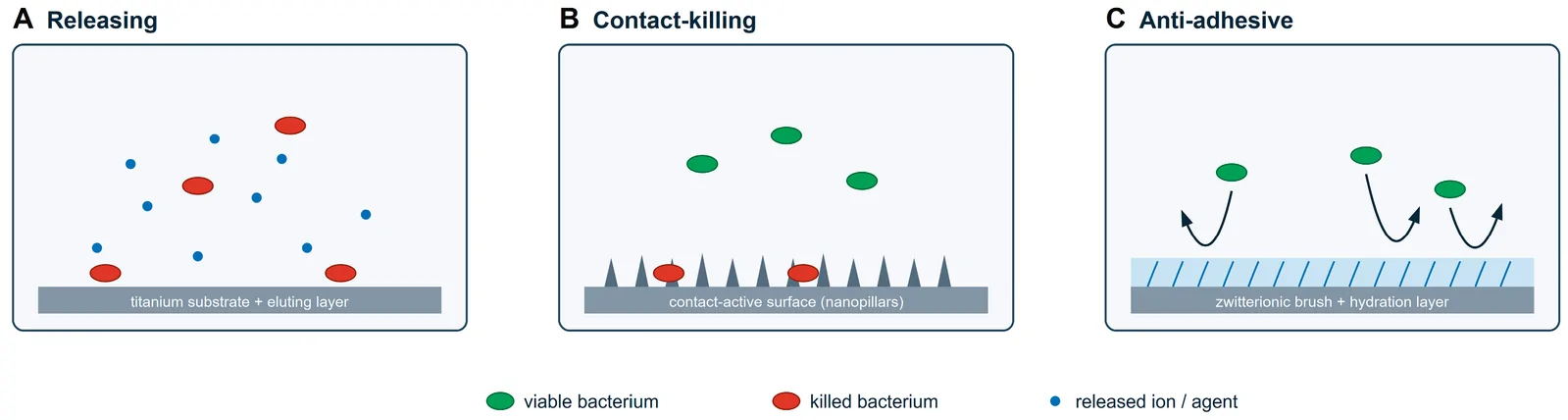
The three antibacterial surface mechanisms and the evaluation each demands. (**A**) Releasing surfaces elute an antibacterial agent that kills both adhered and nearby planktonic bacteria but from a finite, burst-prone reservoir. (**B**) Contact-killing surfaces (for example, bactericidal nanostructures) act only where a cell touches them, so planktonic cells away from the surface survive. (**C**) Anti-adhesive surfaces (hydrophilic or zwitterionic brushes) resist protein adsorption and attachment without any bactericidal agent.

It is useful to organize antibacterial surfaces not by material but by the mechanism through which they act, because, as Section 3 will argue, the mechanism dictates which evaluation method is even appropriate. Broadly, surfaces act by releasing an antibacterial agent, by contact killing on the surface itself, or by resisting adhesion so that biofilm cannot establish [6]. Most real surfaces combine mechanisms, and this is precisely what frustrates single-assay evaluation (Figure 1). Table 1 summarizes the main strategies on this basis, including the translational maturity of each.

### 2.1. Bactericidal Topography and Nanostructuring

Some titanium surfaces kill bacteria physically rather than chemically. Nanoscale protrusions (modeled on the nanopillar arrays of cicada and dragonfly wings) stretch the envelope of an adhering cell beyond its elastic limit until it tears [11]. Because killing is mechanical rather than chemical, it does not select for the resistance mechanisms that undermine antibiotics, which is the main attraction of the approach [21]. On titanium, these features are produced by scalable routes such as hydrothermal or alkaline treatment and plasma etching, the latter yielding a two-tier topography that both resists attachment and ruptures those bacteria that do attach [22,23]. Reported efficacy is geometry- and species-dependent: one hybrid nanowire/nanosheet architecture achieved near-complete kill of Gram-negative *Escherichia coli* (~99%) but only partial kill of Gram-positive *Staphylococcus aureus* (~70%) in the same study [23], following the canonical mechano-bactericidal pattern in which the thinner, less rigid Gram-negative envelope ruptures more readily than the thick, rigid Gram-positive cell wall. The “best” topography therefore depends on which organism is targeted: an early warning that this class will be hard to compare across studies.

A distinctive appeal of bactericidal topography is that the same nanoscale roughness that harms bacteria can favor osteogenic cells: mechano-bactericidal titanium has been shown to support the adhesion, proliferation, and even osteogenic differentiation of stem cells and osteoblast-like cells [22,24]. A recurring limitation, however, is fouling—killed bacteria and debris accumulate on the nanopillars and blunt their activity over time, which has prompted hybrid designs that combine mechanical killing with a bacteria-releasing function to keep the surface active [25].

### 2.2. Antibiotic-Loaded and Eluting Coatings

Coatings that release antibiotics (for example, gentamicin or vancomycin from resorbable carriers) are the most clinically mature strategy, with commercial precedents in fracture fixation—a gentamicin–poly(D,L-lactide)-coated tibial nail is among the few antimicrobial coatings with published clinical data [13]. Their limitation is intrinsic to the release mechanism: a finite reservoir, burst-then-decline kinetics, and the specter of resistance [14]. The burst problem is concrete—conventional antibiotic-loaded bone cement releases most of its drug within days, after which local concentrations can fall below inhibitory levels and may even help select resistant strains; nanocarrier formulations extending release for up to 50 days have been proposed to counter this [26]. Whether a longer release is desirable, however, depends on what it is meant to achieve. The decisive window for implant-associated infection is the peri-operative and early post-operative period in which the surface is colonized before host tissue integrates it, and many carriers are designed deliberately to cover only that window rather than to elute for months; a two-week release is not inferior per se to a fifty-day one. What matters for comparability is therefore not the absolute duration. It is whether a study states the duration it is targeting, the rationale for that target in relation to the healing and colonization timeline, and the concentration maintained relative to an inhibitory threshold. None of the three can be recovered from an elution curve reported without a stated target.

### 2.3. Silver and Metal-Ion/Inorganic Coatings

Silver, applied as nanoparticles, ions, or sputtered films, provides broad-spectrum activity through ion release and oxidative damage and is the most extensively studied inorganic agent in the orthopedic surface literature [8,14]. It is also the inorganic agent that has actually reached patients: silver-coated tumor and revision megaprostheses are in clinical use, and multicenter case series report encouraging infection-control rates in high-risk limb salvage [15,27]. Tellingly for this review, though, the clinical evidence is dominated by retrospective series, and a dedicated review concluded that the benefit of silver coating “could not be confirmed” even as most retrospective data suggested a reduced infection rate [15]; comparability fails even at the clinical endpoint (Section 3.7). The wider inorganic palette (copper, zinc, magnesium) is largely preclinical but advancing: zinc- and magnesium-doped surfaces are pursued as much for their osteogenic as their antibacterial effect, again coupling the two endpoints that Section 4 argues are too rarely measured together [28].

### 2.4. Dual Osteogenic–Antibacterial Surfaces

A growing design goal is to combine antibacterial action with active promotion of bone formation, for instance by co-doping strontium with silver or by loading silver into titania nanotubes [8,16,17]. The osteogenic half of such surfaces is not restricted to inorganic dopants: organic osteoinductive agents (bone morphogenetic proteins, other growth factors, and vitamin derivatives) are increasingly combined with inorganic and organic antibacterial components alike, as in plasma electrolytic oxidation layers that carry an antipathogenic metal-oxide coating together with BMP-2 [29]. Osteogenesis is also not the only second function paired with bacterial killing; surfaces intended to be simultaneously anti-inflammatory or immunomodulatory raise exactly the same evaluation problem, and what follows applies to any dual-function surface. We develop the antibacterial–osteogenic pair as the representative case because it is the best documented, not because it is the only one. These surfaces are the most demanding to evaluate because two competing endpoints must be measured together (Section 4).

### 2.5. Plasma-Based Surface Modification

Plasma and vacuum processes (Table 1) modify chemistry and topography with fine process control and strong coating adhesion. Their appeal for implants is that they can build a functional surface that is metallurgically bonded to the substrate rather than merely deposited on it, and that they can co-deliver antibacterial agents and osteoconductive chemistry in a single step; micro-arc-oxidized titania layers carrying calcium, phosphorus, and silver are a representative example [18]. A magnetron-sputtered silver film outperformed ion implantation and electroplating in one screening comparison [5]. Metal ions are not the only payload and increasingly are not the preferred one: plasma-activated or plasma-oxidized titanium is widely used as an anchoring layer for organic antibacterial agents (conventional antibiotics such as gentamicin and vancomycin and non-antibiotic organic actives) because the plasma step supplies the reactive surface chemistry for attachment without a separate coating [30,31]. That shift is in part a response to the inconclusive clinical record of silver noted in Section 2.3 [15]. Because plasma methods sit at the intersection of surface chemistry and durable adhesion, they are well positioned for translation (and, as Section 3.4 notes, some plasma-derived layers even improve tribocorrosion resistance [18]), yet the supporting antibacterial evidence remains dominated by single-strain in vitro screens [5].

### 2.6. Anti-Adhesive (Non-Fouling) Surfaces

A third mechanistic class neither kills nor releases an agent but simply denies bacteria a foothold, and it completes the release/contact-kill/anti-adhesion triad around which this review is organized. Hydrophilic polymer brushes, most prominently zwitterionic coatings (Table 1), hold a tightly bound hydration layer that resists the protein adsorption on which bacterial attachment depends, reducing adhesion of staphylococci and other organisms without any bactericidal agent [19,20]. Because nothing is consumed, a well-anchored brush can resist biofilm formation over days to weeks, and such coatings have limited bacterial attachment in subcutaneous and transcutaneous implant models [19,20]. Two features bear directly on evaluation. First, most demonstrations are on soft, non-orthopedic substrates (silicone, polyurethane, hydrogels, intraocular lenses) rather than load-bearing titanium, so the durability of the grafted layer under wear and micromotion is largely untested (the same characterization-and-durability gap raised in Section 3.4 and Section 3.5). Second, this class is the starkest case of the mismatch developed in Section 3.2.

## 3. The Evaluation Gap, Level by Level

### 3.1. Two Boundary Conditions: Research Stage and Implant Class

A criticism of evaluation practice is meaningful only if it is addressed to the right stage of investigation, and three stages should be distinguished. In exploratory, discovery-stage work, the object is to establish that a surface has an effect and to understand why; applying several non-equivalent assays is frequently the purpose of the experiment rather than a defect in it, because a mechanism is inferred by triangulating across measurements that do not agree by construction. In preclinical translational work, the object changes: a defined surface on the final device geometry must be shown to perform well enough and durably enough to justify testing in patients, and here non-comparability carries a direct cost, because candidates have to be ranked against one another and against the standard of care. In clinical evaluation, the object is an effect estimate in patients, and comparability depends additionally on a shared outcome definition. The argument of this review is directed at the second and third stages. We do not ask discovery-stage studies to adopt a single protocol; for those studies, the requirement is disclosure (that the assay, strain, inoculum, and metric be reported completely enough for a reader to know what was measured) rather than uniformity. Where the sections that follow criticize heterogeneity, the criticism is that heterogeneity is undeclared and uncontrolled at the point where candidates begin to be compared, not that variety exists in early work. The same distinction bounds the more engineering-oriented requirements introduced later: durability, corrosion, and final-geometry characterization become expectations when a surface is advanced as a translational candidate, not when it is first described.

The second boundary condition is the class of implant. “Orthopedic implant” spans permanent load-bearing joint replacements, temporary or mid-term trauma fixation devices, spinal constructs, and resorbable bone-void fillers. These differ in surface morphology (dense machined or polished surfaces versus porous ingrowth structures), in substrate material (titanium and cobalt–chromium alloys, polymers such as poly-ether-ether-ketone [32], and degradable ceramics or composites), in intended service life (weeks to decades), and in mechanical loading. Each difference changes which evaluation is informative. A durability test built around wear and micromotion is essential for a press-fit arthroplasty surface and largely irrelevant for a resorbable filler. A coating-adhesion standard developed for a dense metal substrate does not transfer unmodified to a porous ingrowth structure, whose internal surface the same measurement cannot reach. An elution profile adequate for a nail intended for removal after fracture union may be inadequate for a permanently implanted bearing. This review is therefore scoped to permanent, load-bearing arthroplasty and reconstruction implants, with periprosthetic joint infection as the clinical endpoint of interest; trauma fixation, spinal, and degradable devices appear only where their evidence illuminates that setting. Readers should not assume that the reporting expectations proposed below transfer item-for-item to those other classes, and the differences just described are precisely why a single evaluation standard spanning every orthopedic implant is unlikely to be workable, whereas a standard defined per surface mechanism within a stated implant class is.

### 3.2. No Single Test Captures All Three Mechanisms

The foundational problem was stated plainly by Sjollema and colleagues: there is no single method or industrial standard that can distinguish antimicrobial surface designs according to all three mechanisms of action, and the available industrial standards each address only one mechanism [6]. The three mechanisms make genuinely different measurement demands. A releasing surface must be characterized by its release kinetics (how much active agent, over what time, against which threshold), so a static endpoint count says little about durability of effect. A contact-killing surface, by definition, only acts where a cell touches it, so a bulk-phase assay that lets bacteria proliferate away from the surface will systematically understate its activity. An anti-adhesive surface is not meant to kill at all, so a viability assay is the wrong instrument entirely; adhesion and biofilm-formation assays are required. An assay chosen for one mechanism applied to a surface acting by another is therefore not merely imprecise, it can be qualitatively misleading, which is exactly the failure documented in Section 3.3. Because investigators need to show something, the standards have been modified ad hoc over the years, “adding to the myriad of methods” and making results progressively harder to compare [6]. Sjollema and colleagues framed adequate, standardized in vitro data as a prerequisite gate for justifying animal experiments and clinical trials, which means the evaluation gap is not a downstream reporting nuisance but an upstream barrier to translation [6].

### 3.3. In Vitro Assay Heterogeneity

Below the level of “which mechanism,” the in vitro literature is heterogeneous in almost every operational choice. A systematic review of strontium/silver titanium coatings classified the antibacterial assays used into three broad and non-equivalent families (microscopy-based, agar-based zone-of-inhibition or colony counting, and turbidity/optical-density growth) and found that a minority of studies relied on a single method, while biofilm, when assessed at all, was usually described qualitatively rather than quantified [8]. Strains cluster around *Staphylococcus aureus* but vary in species, reference versus clinical origin, and inoculum, and incubation times and quantification metrics differ throughout [8,14].

The problem is not only that different studies choose different endpoints but that even a single endpoint can be measured in ways that do not agree. Implant-associated biofilms have been quantified in parallel by LIVE/DEAD confocal microscopy, plate counting after ultrasonic or enzymatic detachment, resazurin and ATP-bioluminescence viability assays, and crystal-violet staining. The methods diverged sharply in what they captured, in reliability, and in whether they suited initial adhesion or mature biofilm. The authors concluded plainly that no single method serves all purposes and that the choice must follow the specific question [33]. The mismatch is not merely academic: crystal-violet staining, one of the most widely used biofilm assays, proved unusable on a clinically relevant graft surface because of high background, and the correlation between ATP-based and colony-count readouts was itself strain- and surface-dependent [34]. Two laboratories measuring “biofilm” on the same material with different but individually accepted assays can therefore obtain results that cannot be placed on a common scale: a within-endpoint version of the same comparability failure that operates across endpoints.

**Figure 2:**
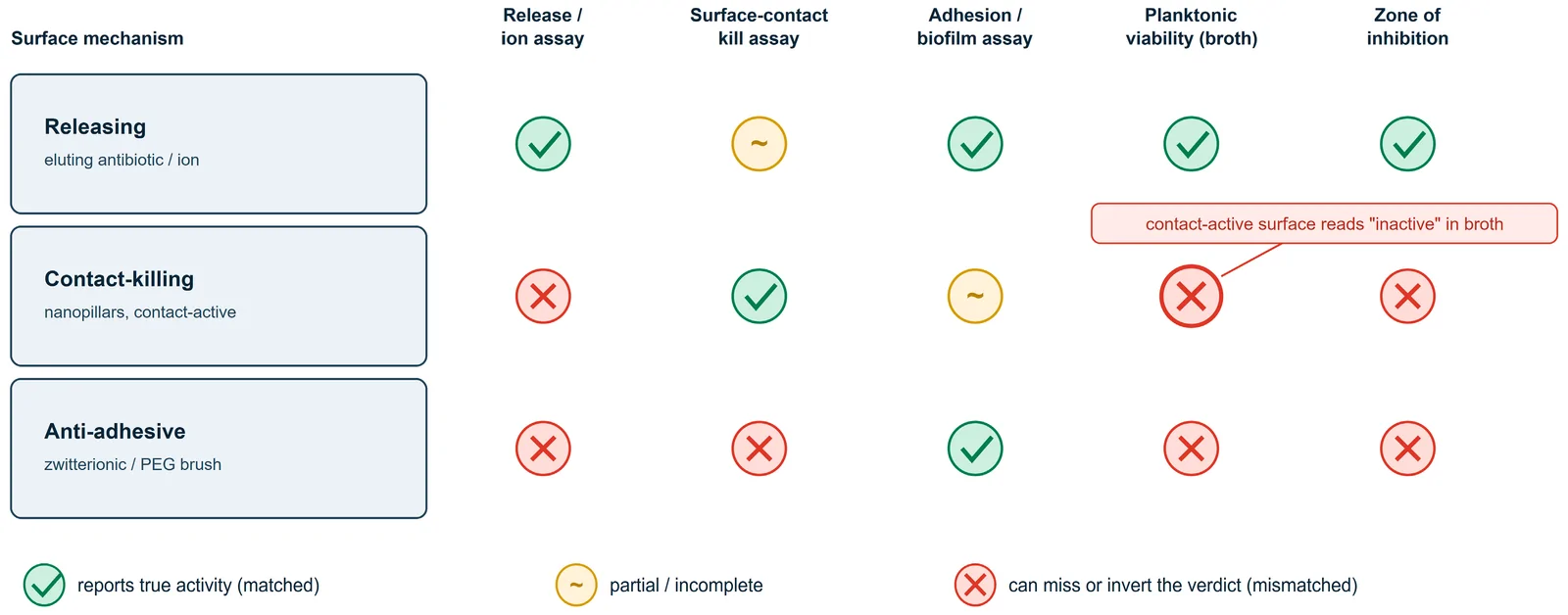
Mechanism–assay matching. For each surface mechanism (rows), an assay (columns) either reports the surface’s true activity (matched), gives only a partial picture, or can miss or invert the verdict (mismatched). Adhesion and biofilm readouts share one column: for a contact-killing surface, the biofilm half reports the effect, whereas an adhesion count alone can under-report it, because killed cells may remain adherent.

That this heterogeneity is decisive, not incidental, has been shown directly. In the demonstration designed to test it, Campos and colleagues put the same antimicrobial films through four protocols: a modified ISO 22196 [35], ASTM E2149 [36], a dried-droplet method, and a transfer method, with most of the films tested in three of the four. They concluded that efficacy “cannot be easily and reproducibly compared” across them [4]. Their test surfaces were commercial hard-surface films rather than implant coatings, but the protocol-dependence they expose is generic. A single-study example illustrates how this plays out mechanistically: a magnetron-sputtered silver coating produced near-complete kill in a membrane-contact assay yet showed no statistically significant activity in a planktonic co-culture assay against *S. aureus* [5]. Because those two assays interrogate different things (surface contact-killing versus bulk-phase planktonic viability), a contact-active coating can legitimately read as potent in one and inert in the other, so a test chosen for convenience rather than mechanism can decide the outcome. Figure 2 sets out which assays report and which misread each mechanism.

### 3.4. Mechanical, Adhesion, and Corrosion Testing

An antibacterial surface that delaminates, wears, or corrodes in service is not antibacterial for long, yet coating durability is evaluated far less consistently than antibacterial activity, and often not at all. Mature standards do exist for coating mechanics and electrochemical stability on metallic implants: ASTM F1044 for shear strength [37] and ASTM F1147 for tensile (normal) adhesion of coatings to metal substrates [38], ISO 13779-4 for the adhesion of hydroxyapatite coatings [39], and ASTM F2129 for cyclic potentiodynamic corrosion susceptibility of small implant devices [40]. These standards are themselves proprietary and paywalled, which limits independent scrutiny of how they have been applied; freely available descriptions of what they require and of the characterization data expected for coated orthopedic devices are given in the corresponding regulatory guidance [41] and, for ISO 22196, in the comparative analysis discussed in Section 5 [42]. The problem is twofold. These standards were written for osseointegrative and structural coatings or for the metallic device itself, not for thin antibacterial films or ion-releasing layers, so their applicability is uncertain, and, in practice, antibacterial-surface studies frequently omit adhesion, wear, and corrosion testing altogether. Authors of dual-function studies have themselves acknowledged not testing coating adhesion strength or production/transport stability [17]. The mechanical and electrochemical durability of antibacterial surfaces is therefore an evaluation dimension that is available in principle but neglected in practice.

Where durability is examined, the tribocorrosion literature shows both why it matters and how differently it is done. In load-bearing service, the implant experiences wear and corrosion simultaneously, and their interaction, not either alone, governs degradation: sliding removes the passive oxide film, exposing fresh metal that corrodes, so that a galvanic couple forms between worn and unworn regions and drives wear-accelerated corrosion [43]. This directly threatens an ion-releasing antibacterial surface, whose function depends on a controlled, intact surface chemistry that mechanical damage can alter unpredictably. Yet the way tribocorrosion is assessed varies widely: studies differ in the counter-body and load, in whether tests run at open-circuit or controlled potential, in the simulated body fluid used, and in how they partition total material loss into wear-driven and corrosion-driven fractions [18,43]. Some antibacterial coatings deposited by plasma routes have been shown to improve tribocorrosion resistance: micro-arc-oxidized titania films, including silver-bearing variants, can shift the dominant regime toward pure wear and away from wear–corrosion synergy [18]. The same coupling is now being engineered deliberately: in the wider protective-coatings literature, metal-organic-framework layers are designed to deliver antimicrobial action and corrosion resistance together [44], so that the two properties become functions of one surface chemistry and cannot be evaluated in isolation from each other. Because the test protocols themselves are not standardized, however, such durability results are no more comparable across studies than the antibacterial ones. Durability and antibacterial evaluation thus fail to be comparable for the very same reason, at two different ends of the same device.

### 3.5. Surface Characterization

Claims about why a surface is antibacterial rest on knowing what is actually on it. In several studies, the surface chemistry of the finished, complex-geometry implant is not measured directly (for example, by X-ray photoelectron spectroscopy or time-of-flight secondary-ion mass spectrometry), and efficacy is instead inferred indirectly from simulations and functional assays, an omission the authors have disclosed as a limitation [45]. The gap matters for two reasons. The first is mechanistic attribution: if a surface is antibacterial but its composition, oxidation state, and ion-release profile on the actual device are unknown, the observed effect cannot be confidently assigned to the intended agent rather than to a processing artifact or contaminant. The second is reproducibility: coating chemistry can vary between batches and, critically, between a flat test coupon and a porous, curved implant, so characterization performed only on an idealized witness sample may not describe the device that is actually implanted. Direct characterization on the final geometry is what links a functional result to a defined material state; without it, both the mechanism and the manufacturability of the surface remain assumptions rather than measurements.

### 3.6. In Vivo Model Heterogeneity

Animal models of PJI range from simple transcutaneous wires to load-bearing proximal-tibial implants, and this variety in species, implant, bacterial strain, and readout limits comparison across studies [46]. The dimensions of variation are numerous and consequential. They include the host species and its immune competence; whether the implant bears load and osseointegrates, as a human prosthesis does, or merely sits in soft tissue; the inoculation route and dose; the bacterial species and whether a laboratory reference or a clinical strain is used; and the readouts chosen to define infection. Each of these can shift the apparent performance of a surface, so a coating that looks protective in a non-load-bearing pin model may behave differently at a weight-bearing, osseointegrated interface where micromotion, wear debris, and a mature biofilm all come into play. The field has begun to converge on what a clinically representative model requires—similarity to human immune and musculoskeletal systems, a weight-bearing implant that reproduces the periprosthetic environment, a clinically relevant strain, and readouts that quantify biofilm, bacterial load, and host response [47]. Standardized examples now exist, including a rabbit model with scanning-electron-microscopy biofilm quantification on the tibial explant [47] and a load-bearing three-dimensionally printed Ti-6Al-4V murine model that permits quantitative periprosthetic bacterial-load analysis [48]. These are advances, but they are not yet a single agreed baseline, and most antibacterial-surface papers either use idiosyncratic models or stop at in vitro testing [14], so even the preclinical evidence that does exist is difficult to pool.

Two qualifications belong here. Model variety at the earliest stage is not in itself a defect. Implantation in soft tissue cannot reproduce a periprosthetic interface, but it yields usable information about the local inflammatory response to a material and can eliminate candidates before a bone model is justified, and the requirements for such local-effect testing after implantation are themselves standardized [49]. Nor does in vitro cytocompatibility reliably predict the in vivo local response, which is part of why an in vivo stage is necessary rather than a formality. What the comparability argument asks is therefore not that every study adopt the same model but that the model chosen be justified against the question being asked and reported completely enough for a reader to know what it did and did not reproduce.

Not all of these dimensions are likely to matter equally. The evidence does not yet support a formal ranking, but a tentative ordering is more useful to a reader than a flat list, and two variables plausibly dominate. The first is whether the implant is loaded and osseointegrated, because micromotion, wear debris, and a mature periprosthetic interface alter both the bacterial niche and the host response and because a surface can only be shown to survive service conditions in a model that imposes them. The second is inoculum dose, which sets the event rate a study is able to detect. The rabbit model examined in Section 7 deliberately used three inoculation doses (10^3^, 10^4^, and 10^5^ CFU) because published infection rates in comparable models differ with dose; the earlier series that it cites reported 40% at 10^3^ versus 70% at 10^5^ CFU [50]. Two studies using different doses may therefore differ in apparent protection without differing in the surface. Host species and immune competence follow; strain provenance, reference versus clinical isolate, appears to affect the size of an observed effect more than whether an effect is observed at all. We offer this ordering as a hypothesis for the inter-laboratory trials proposed in Section 9, not as an established hierarchy.

### 3.7. Clinical Endpoint Heterogeneity

**Figure 3:**
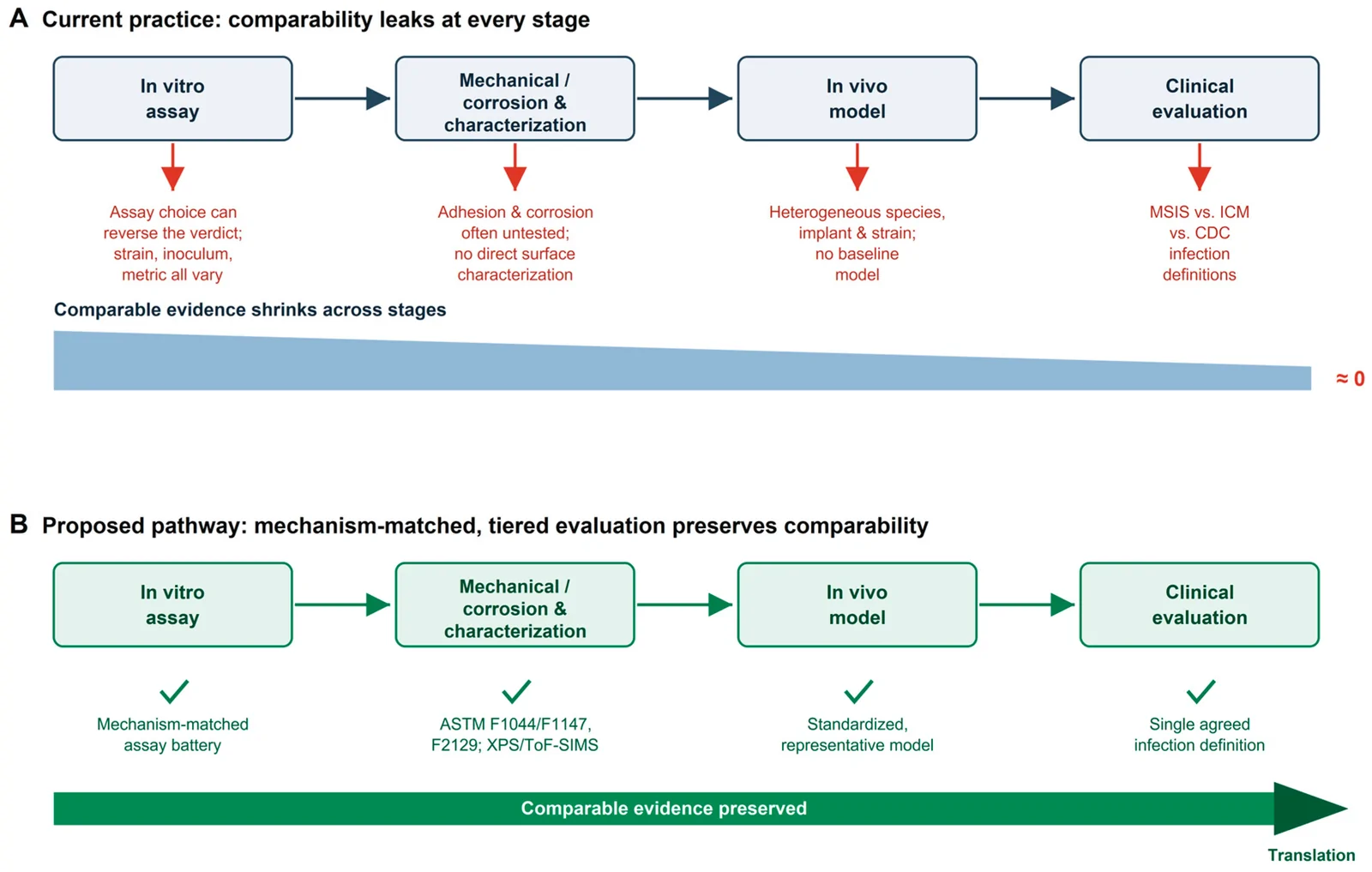
Where comparability is lost and how a tiered pathway preserves it. (**A**) Current practice: each stage of the pipeline introduces its own source of non-comparability (red), and the tapering wedge indicates that the pool of mutually comparable evidence shrinks stage by stage, approaching zero at the point of clinical comparison. (**B**) Proposed pathway: the same stages, each now carrying the mechanism-matched, tiered minimum requirement developed in Section 7 and itemized in Table 2, under which comparable evidence survives to support translation.

At the clinical end of the pipeline, comparability fails for a more basic reason: studies do not agree on what counts as an infection. A 2026 network meta-analysis of antimicrobial implant-surface coatings covered 26 studies and 3592 patients. Its included studies were split across several competing definitions: the Musculoskeletal Infection Society (MSIS) criteria, the International Consensus Meeting (ICM) criteria, the Centers for Disease Control and Prevention (CDC) definitions, the European Bone and Joint Infection Society (EBJIS) definition, and, in some cases, unvalidated criteria. The authors identified the absence of a standardized infection definition as a critical barrier to comparability, compounded by heterogeneity in study design, follow-up duration, and reporting and by largely unadjusted data [9]. Even this recent synthesis, which reported lower infection odds with certain coatings (e.g., DAC-hydrogel OR 0.10, gentamicin OR 0.27, silver OR 0.67), explicitly cautioned that definitional inconsistency and residual confounding limit ranking reliability. When the outcome itself is defined differently from study to study, pooling and ranking of surface technologies become unreliable no matter how good the underlying materials are. The problem is compounded by how little clinical evidence exists at all: a clinical review of antimicrobial-coated trauma and orthopedic implants could identify only four coating technologies with any clinical data, across nine studies and 435 patients, of which just two were case-control studies and the remainder uncontrolled case series [13]. Silver-coated megaprostheses, the most extensively used inorganic coating in high-risk revision and tumor cases, still rest largely on retrospective series whose infection-rate benefit “could not be confirmed” in a dedicated review [15]. When the highest-tier evidence is this sparse and this heterogeneous in design, definition, and follow-up, a genuine comparative ranking of surfaces is out of reach. Taken together, comparability is eroded at every successive stage of evaluation (Figure 3A).

## 4. The Antibacterial–Osseointegration Trade-Off and Coherent Dual-Function Evaluation

A recurring tension runs beneath the whole field: the same properties that kill bacteria can harm bone-forming cells. The trade-off is often characterized as near-fundamental, on the grounds that many bactericidal agents struggle to achieve acceptable antibacterial activity within a cytocompatible dose range [8]. Concretely, one study reported that a silver-treated surface reduced osteoblast-like cell attachment by more than 60% at the concentration and assay tested [45]; the authors of that study regarded the effect as desirable for a plate intended to be removed, but it measures the cost when osseointegration is the goal. The biological reason is that several of the mechanisms that make an agent bactericidal are not bacteria-specific. Released silver ions and the reactive oxygen species they generate damage membranes, proteins, and DNA in eukaryotic cells through the same mechanisms that act on bacteria, generally at somewhat higher doses; the dose that clears bacteria therefore sits below the dose that impairs osteoblast adhesion and mineralization, but often uncomfortably close to it. The practical consequence is the concept of a therapeutic window: a usable surface must release enough active agent to exceed the minimum bactericidal concentration yet stay below the cytotoxic threshold, and that window can be narrow, agent-specific, and dependent on release kinetics rather than total loading. Design strategies exist to widen it—combining strontium with silver, for instance, can partly counterbalance silver’s cytotoxicity while preserving antibacterial effect [8,16], and zinc- or magnesium-based surfaces are pursued partly because they are actively osteogenic rather than merely tolerated [28]. Some work additionally considers the host response, tuning surface chemistry to steer macrophage polarization toward a pro-healing phenotype [28], although this immunomodulatory dimension is not yet evaluated consistently enough across studies to serve as a routine reporting item, and Table 2 accordingly treats it as emerging rather than required. However, the more important point for this review is how rarely these endpoints are evaluated together and coherently. Part of the reason is structural: antibacterial and osteogenic assays are often the province of different laboratories, cell types, and timeframes, so pairing them in one study on one surface geometry is the exception rather than the rule.

A few studies illustrate what coherent dual-function evaluation looks like. In one, silver-loaded titania nanotube arrays kept silver release (maximum 0.3033 ppm) inside a therapeutic window bracketed by a bactericidal threshold (~0.1 ppm) and a cytotoxic threshold (~1.6 ppm), while simultaneously demonstrating sustained antimicrobial activity, absence of MC3T3-E1 cytotoxicity, enhanced early cell adhesion, and up-regulated osteogenic markers [17]. A magnetron-sputtered micron-silver coating similarly paired anti-biofilm function with osteogenic marker expression at a low, sustained release (10–421 ng/mL over 90 days) and non-cytotoxic fibroblast viability by the ISO 10993-5 standard [5,51]. The value of these examples is methodological, not the specific numbers: they measure both endpoints and interpret antibacterial dose explicitly against a toxicity threshold—the practice this review advocates. The thresholds themselves are single-study values and should not be read as benchmarks (other work places silver cytotoxicity above 2 ppm), and the release windows are in vitro, not validated in vivo [5,17]. What makes them instructive is precisely that they are uncommon: many studies still report a single endpoint, leaving the trade-off asserted rather than demonstrated.

## 5. Standards: What Exists and Why They Fall Short

The industrial standards most often invoked each interrogate essentially one mechanism [6,7]. ISO 22196 (and the closely related JIS Z 2801 [52]) was written for contact activity on flat, non-porous industrial surfaces. ASTM E2149 instead agitates the specimen in inoculum under dynamic contact conditions and, because the inoculum never rests on the material, registers only release-based activity. Their design assumptions expose the mismatch. ISO 22196 sandwiches a defined inoculum under a thin film on a flat coupon at fixed humidity for 24 h; it presumes a two-dimensional, non-porous, non-absorbing surface and takes a planktonic inoculum, rather than an established biofilm, as the target. An orthopedic implant surface violates these premises: it is three-dimensional and often deliberately porous, and the clinically relevant challenge is a mature biofilm, not a planktonic film. The method does register release-based as well as contact killing, since both act within that liquid film, so the mismatch is one of geometry and target state rather than of kill mechanism, but it is severe enough that a result on a flat coupon need not predict behavior on the real device. A dedicated review of standardized antibacterial material testing methods reached the same conclusion—no existing standard test simultaneously accommodates the releasing, contact-killing, and anti-adhesion mechanisms that real implant surfaces combine [7]—and this is the same gap that Sjollema and colleagues identified at the level of mechanism [6].

A further structural problem is that the standards that are mandatory for implants address a different question. Regulatory biocompatibility testing under the ISO 10993 series evaluates whether a material is safe (cytotoxicity, sensitization, ion-release toxicity), not whether, or how well, it kills bacteria. There is, in effect, a required standard for the toxicity half of the therapeutic window (ISO 10993-5 cytotoxicity is routinely cited, as in the magnetron-sputtered silver example of Section 4 [5]) but no equally established, implant-specific standard for the antibacterial half. Efficacy is thus assessed by whatever laboratory assay the investigators select while safety is assessed against a fixed international standard: an asymmetry taken up in Section 6.

Standards are evolving, but not toward implant-specific unification. The newly published ISO 7581:2023 [53] offers a more realistic non-porous surface test than ISO 22196:2011 [35], yet its own reproducibility is limited by the low application volume and by strain-dependence, so that protocol adaptations remain necessary [42]. Tellingly, even this newest surface standard is still written for non-porous surfaces: the very geometry an implant does not have. In short, the current generation of surface standards does not yet provide the mechanism-spanning, implant-relevant, reproducible test that the field has been calling for since 2018 [6,7], and there remains no consensus minimum-reporting framework specific to antibacterial implant surfaces.

## 6. Regulatory Expectations: Harmonized Safety, Discretionary Efficacy

A framework meant to support translation has to be legible to the bodies that authorize translation, and the regulatory position is instructive because it reproduces, rather than resolves, the asymmetry described in Section 5. In the United States, an orthopedic implant carrying an antibacterial surface reaches the market through premarket notification (510(k)), premarket approval, or, where no suitable predicate exists, the de novo classification route, which is the pathway that novel antibacterial implant coatings have in fact taken. What the agency does not do is measure antibacterial performance against a recognized efficacy standard, because none exists for implant surfaces. In vitro antibacterial data alone are not accepted as sufficient support for an antibacterial claim; animal or clinical evidence is recommended in addition to bench testing. Authorization turns instead on a device-specific risk assessment, because as of 2024, no guidance document or recognized standard had yet established special controls for antimicrobial orthopedic devices. The concerns such controls would have to address (uncontrolled or insufficient release, loss of a stable release profile across the infection period, interference with bone growth and osseointegration, and maintaining antibacterial activity without impairing biocompatibility or mechanical stability) are still framed as unmet development challenges rather than as codified requirements [54]. That list is close to the content of Table 2, which is not a coincidence: these are also the questions a reader of the primary literature currently cannot answer.

In the European Union, the same asymmetry takes a different form. Where the antibacterial function is delivered by a medicinal substance (the antibiotic-loaded coatings and cements of Section 2.2), the device falls under Rule 14 of Annex VIII to Regulation (EU) 2017/745 and is classified as Class III. Conformity assessment then requires the quality, safety, and usefulness of that substance to be verified by analogy with the requirements applying to medicinal products, with consultation of a competent authority for medicinal products [55]. Biological safety is addressed through the ISO 10993 series, including the recently revised requirements for local effects after implantation [49]. None of this constitutes a standardized measure of antibacterial efficacy, and none of it requires efficacy to be reported in a form that permits comparison with a competing surface. Safety is harmonized; the evaluation of efficacy is left to the sponsor. The point is visible in the guidance itself. FDA issued draft guidance in 2024, still not final, on metallic and calcium-phosphate coatings for orthopedic devices. That scope covers metallic, calcium phosphate, and dual coatings alike, so a sputtered silver film falls within it as a metallic coating. Its stated scope, however, is the characterization of the coating and of how it is made, not the demonstration of antibacterial performance [41].

Two consequences follow. First, the comparability gap is not a publishing artifact that regulation corrects downstream: because each device is assessed against its own risk profile rather than against a common efficacy benchmark, neither the literature nor the regulatory record accumulates into a comparable evidence base. Second, and more constructively, the minimum dataset proposed here is close to what a sponsor must assemble anyway. Declaring the intended mechanism, matching the assay to it, reporting strain, inoculum, and metric, demonstrating coating adhesion and corrosion behavior on the final geometry, and clearing a clinically representative in vivo model before a clinical claim is made are all elements that appear, in one form or another, in a risk-based submission. Adopting them as reporting expectations in the literature would therefore align published evidence with evidence that is already being generated, rather than impose an additional burden. The limits of that alignment should be stated plainly: what is proposed here is a reporting and comparability instrument, not a regulatory pathway, and nothing in it should be read as anticipating what any agency would accept.

## 7. Toward Comparability: A Tiered Evaluation Framework

The pattern across Section 3, Section 4 and Section 5 is consistent: at every level, the field has methods but not shared methods, and comparability is the casualty. A constructive response does not require inventing new assays so much as agreeing on how to select, combine, and report existing ones. We therefore propose three coupled elements, summarized as a minimum-reporting checklist in Table 2 and as a staged pathway in Figure 3B. The three do different work and should not be conflated. Mechanism-matched assay selection is the element that most directly improves comparability, because it stops studies from measuring incommensurable things. The reporting checklist primarily improves transparency and auditability, letting a reader see whether two studies are comparable rather than making them so. The staged gate organizes when each type of evidence is required. Transparent reporting is a precondition for comparability, not a guarantee of it. The checklist is also deliberately stage-conditional rather than uniform. Three of the demands are engineering expectations appropriate to a translational candidate: that a study characterize the final device geometry, that it test coating adhesion and corrosion, and that it use a clinically representative infection model. Applying them to exploratory work would misdescribe what that work is for. The corresponding items are therefore required from stage S2 onward, while what is asked of discovery-stage studies is complete reporting of the assay, strain, inoculum, and metric they did use (Table 2, final column). Method plurality in early work is not the problem this review identifies. The problem is that plurality persists undeclared into the stage at which candidate surfaces are ranked against one another, so that differences in reported performance cannot be separated from differences in how performance was measured. Each checklist item derives from a comparability failure documented in Section 3, Section 4 and Section 5, so the proposal is traceable rather than free-standing.

It should be read, however, as a concrete starting point for a community consensus process, not as a finished or authoritative standard: a checklist proposed by one group has no more authority than the ad hoc practices this review criticizes until it is tested and refined. Establishing it would require the same machinery used to develop other health-research reporting guidelines such as CONSORT, PRISMA, ARRIVE, and SANRA. That machinery has three parts: a multi-stakeholder Delphi process involving materials scientists, microbiologists, orthopedic clinicians, regulators, and journal editors; pilot application to published studies to confirm that it discriminates comparable from non-comparable work, which we begin below; and inter-rater reliability testing [56]. We offer the framework here expressly to make that process concrete and testable, not to pre-empt or claim ownership of it.

### 7.1. Mechanism-Matched Assay Selection

Studies should first declare the intended mechanism(s) of their surface and then select the assay battery that interrogates them—release quantification for eluting surfaces, contact-kill assays for contact-active surfaces, and adhesion/biofilm assays for anti-adhesive surfaces—rather than defaulting to whichever protocol is convenient.

### 7.2. A Minimum-Reporting Checklist

Cross-study comparison would be transformed by consistent reporting of a small set of items, set out in Table 2: what the surface was tested against and under what conditions, with what metric, and, from the translational stage, with what evidence of durability, direct characterization, and in vivo performance.

### 7.3. A Staged Evaluation Gate

Finally, evidence should progress through defined tiers—in vitro efficacy with mechanism-matched assays → mechanical/adhesion/corrosion and direct characterization → a clinically representative, standardized in vivo PJI model [47,48] → clinical evaluation using a single agreed infection definition [9]—with each tier acting as a gate to the next. The point of the gates is to stop a surface from being promoted on the strength of evidence from one tier alone: a striking in vitro kill result should not, by itself, license a clinical claim if durability, characterization, and a representative in vivo model have not been cleared. This mirrors the translational logic Sjollema and colleagues implied [6] and would let reviewers and regulators locate any given surface on a common ladder, rather than comparing surfaces that were never evaluated on the same terms.

### 7.4. Pilot Application to Published Studies

The checklist above is a proposal, and a proposal of this kind is testable in one obvious way: applied to published work, does it separate studies whose results can be placed on a common scale from studies whose results cannot? We therefore applied it retrospectively to a sample of the recent literature. Open-access primary studies of antibacterial titanium or titanium-alloy surfaces for bone-anchored implants published between 2022 and 2025 were identified in PubMed, and the first thirteen eligible records were scored, with deliberate coverage of all three mechanism classes; the search string, eligibility criteria, and per-item scoring notes are given in the Supplementary Material. Restricting the sample to open-access articles was a methodological choice rather than a convenience, because every scoring decision can then be checked by a reader against the same sentences we read. Each study was first assigned a stage as defined in Section 3.1; each item was then scored as reported, partially reported, or not reported, and marked not applicable where the study’s stage, or the absence of a dual-function claim, did not require it. Scoring was performed by the authors and has not been repeated independently, so the inter-rater reliability that a community version of this checklist would require remains untested.

**Figure 4:**
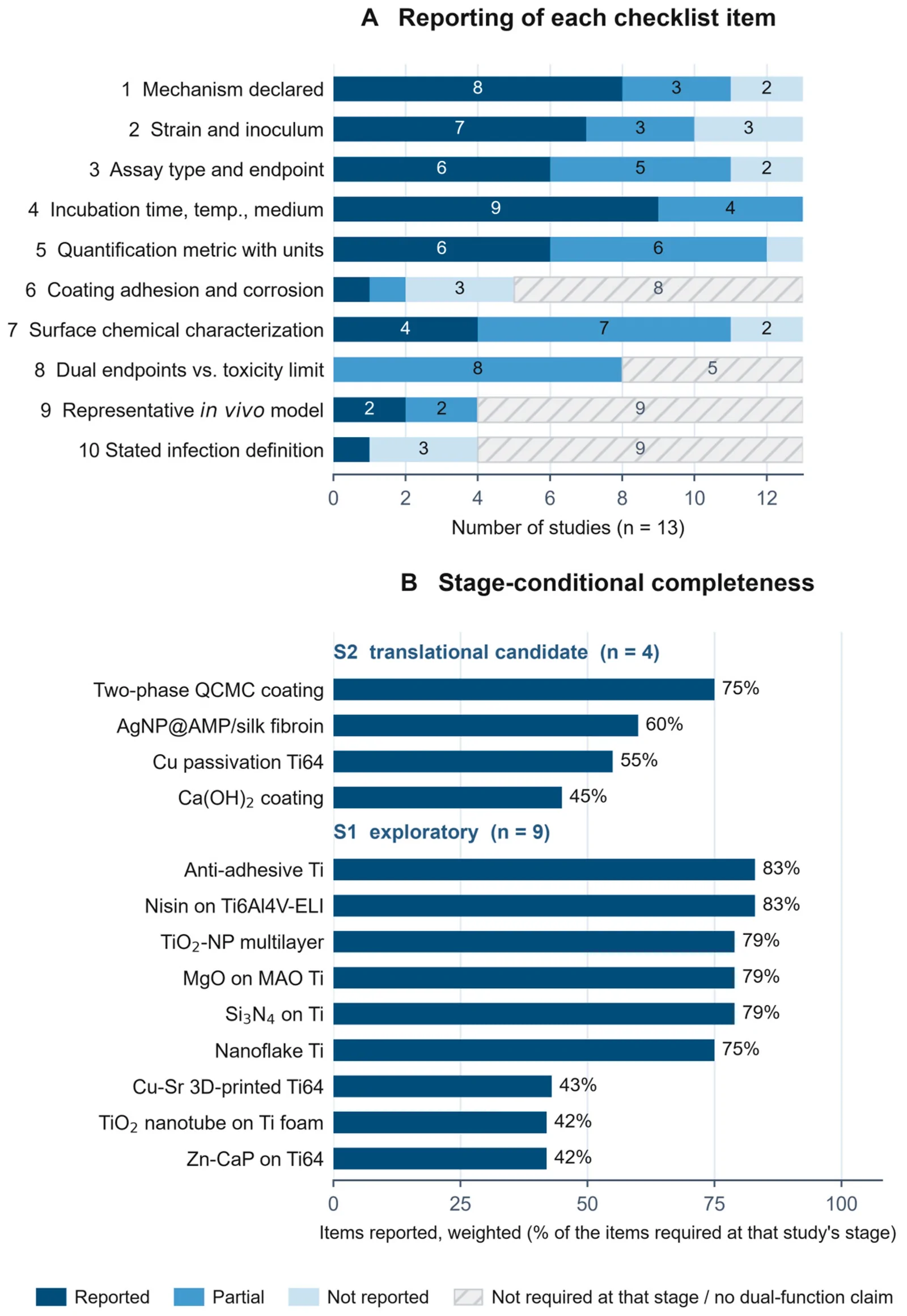
Pilot application of the checklist. (**A**) How each of the ten items was reported across the thirteen studies. (**B**) Weighted completeness per study, computed only over the items required at that study’s stage, scoring a reported item 1 and a partially reported item 0.5. S1 and S2 studies are grouped rather than ranked together because the two groups are assessed against different numbers of items; the scores are therefore comparable within a stage and not across stages.

Two results matter. The first is that the instrument discriminates rather than condemning uniformly (Table 3, Figure 4). Incubation conditions were fully reported by nine of the thirteen studies, so the individual items are plainly achievable, but no study reported every item required at its stage, and one item (antibacterial dose interpreted against a stated cytotoxicity threshold) was reported by none. Low scores therefore reflect absent reporting rather than an unattainable standard. Weighted completeness ranged from 42% to 83% among the exploratory studies and from 45% to 75% among the four studies advancing a translational candidate. The second result is that the failures are not in the same place. The study with the most complete durability data, coating adhesion and potentiodynamic corrosion both measured, did not identify its bacterial strains or state an inoculum in colony-forming units, whereas the only study combining a load-bearing in vivo model with a prospectively stated infection definition (a study-specific composite score rather than a recognized standard) performed no surface chemical characterization at all. Because different studies stop short at different tiers, their reported efficacy figures are not commensurable: this is the comparability leak of Section 3 and Section 5 observed directly rather than argued.

Two gaps were close to universal. Coating adhesion and corrosion were both reported by a single study, which did so although its stage did not require them. Of the four studies that had already proceeded to animal experiments, one reported adhesion alone, and three reported neither. No study interpreted its antibacterial dose against an explicitly stated cytotoxicity threshold, although eight reported osteogenic or cytocompatibility endpoints alongside antibacterial ones. The two halves of a dual-function claim were measured but never quantitatively related to each other (Section 4). The exercise also corrected the checklist. Item 7 originally required characterization on the final device geometry from the preclinical stage, which left no way to record that two studies performed no surface chemical analysis of any kind; the item now distinguishes characterization of any surface, expected from S1, from characterization of the final geometry, expected from S2. We offer this pilot as evidence that the instrument behaves as intended on real papers, not as an estimate of the field’s reporting quality. Thirteen relevance-ranked open-access articles are a convenience sample, single-rater scoring is provisional, and, most importantly, the score measures what a study reported, not the quality of the science it did.

## 8. Transferable Lessons Between Dental and Orthopedic Implant Surface Science

Although this review is orthopedic in focus, the surface science it examines is largely shared with dental implantology, and the exchange runs both ways. The dominant substrate (titanium and its alloys), the leading antibacterial agents (silver, strontium, and related ions), and the plasma and sol–gel processing routes are common to both fields, and the systematic-review evidence on strontium/silver coatings in fact spans dental and orthopedic titanium together [8]. The antibacterial-versus-osseointegration trade-off and the dual-doping strategies used to manage it are field-independent design principles [8,16]. Dental implantology, with its long history of quantitative surface characterization and osseointegration assessment, offers orthopedics a mature template for standardized surface evaluation; orthopedics, in turn, contributes rigorous load-bearing mechanical and in vivo infection models [47,48].

Two specific transfers deserve naming because they concern exactly the two levels at which this review found comparability weakest. The first is the clinical case definition. Implant dentistry faced the problem catalogued in Section 3.7: competing definitions of peri-implant disease that rendered prevalence estimates and treatment comparisons incommensurable. It addressed the problem through a multi-stakeholder consensus workshop, which issued agreed-upon case definitions for peri-implant health, mucositis, and peri-implantitis, with explicit thresholds and provisions for the common situation in which baseline records are unavailable [69]. Those definitions are still debated at the margins, and adoption has been uneven. That is what makes the example useful rather than decorative. It shows that field-wide agreement on an outcome definition is achievable by deliberate process rather than by waiting for convergence and that such agreement is a floor for comparability rather than a final answer. Orthopedics possesses the constituent parts (the MSIS, ICM, CDC, and EBJIS criteria) but has not converged on which of them should be used when an antibacterial surface is evaluated, and the network meta-analysis discussed in Section 3.7 quantifies the cost of that non-convergence [9].

The second transfer concerns surface description. Implant dentistry adopted a shared vocabulary for surface topography early, reporting height and hybrid roughness parameters over defined measurement scales and grouping surfaces into agreed roughness categories, which made it possible to relate bone response to surface class across studies from different laboratories [70]. Antibacterial orthopedic surface papers frequently describe topography qualitatively or report a single parameter measured over an unstated scale, which forecloses the same kind of synthesis: the gap that item 7 of Table 2 addresses. The exchange is not one-directional. Orthopedics contributes what dentistry largely lacks: load-bearing mechanical and tribocorrosion testing appropriate to a joint replacement, and in vivo periprosthetic infection models in which the implant is loaded and osseointegrated [47,48]. The case for defining evaluation standards at the level of the surface mechanism rather than the anatomical site rests on this shared substrate and shared agent chemistry; it is not a claim that anatomical site is unimportant. Site determines the loading regime and the relevant durability tests, and for large bone implants, in particular, it determines whether bulk and structural properties, rather than the surface, dominate performance. Those factors lie outside the scope defined in Section 3.1, and a surface-level reporting standard is complementary to, not a substitute for, evaluation of the device as a whole.

## 9. Conclusions and Future Directions

The antibacterial-surface literature for orthopedic implants is rich in materials and poor in comparability. Because efficacy is evaluated with mechanism-mismatched, non-standardized methods [4,5], the field cannot presently rank its own strategies or move them efficiently toward the clinic. Invention is not finished (no surface yet reliably prevents load-bearing PJI), but the rate-limiting step has increasingly become evaluation rather than invention.

Several directions would move the field toward comparability. First, the checklist of Table 2 needs to be developed into a genuine community standard through the multi-stakeholder consensus process described in Section 7; the pilot reported there tests its discrimination but not its inter-rater reliability [10,56]. Second, the field would benefit from inter-laboratory round-robin studies and shared reference surfaces, in which the same materials are tested by multiple laboratories under defined protocols; such ring trials are how reproducibility has been established in other measurement-dependent fields and would quantify, rather than merely assert, how much assay choice drives the reported outcome. Third, biofilm quantification in particular needs harmonization, given that even a single endpoint is measured by non-equivalent methods [33,34]. Fourth, standardized, clinically representative in vivo PJI models [47,48] should become the expected gate before clinical claims, and journals and regulators could accelerate adoption simply by requiring mechanism-matched assay selection and complete reporting as a condition of publication or submission. Fifth, resistance-emergence itself deserves to be treated as an evaluation dimension rather than an afterthought: contact-active and eluting surfaces differ in their theoretical propensity to select resistant organisms, yet few studies assess it, and the frequently cited resistance-independence of purely mechanical killing is asserted more often than it is tested over clinically relevant exposures. Resistance pressure is also being addressed by strategies pursued in parallel with surface engineering, such as host-directed bacterial immunotherapy, which fall outside the scope of this review but face the same prerequisite: a mechanism-based taxonomy before candidates can be compared [71]. Finally, as automated and AI-assisted screening expands the throughput of candidate surfaces, the case for a shared evaluation standard becomes stronger, not weaker: higher volume without comparability would only deepen the present problem.

Until antibacterial surfaces are evaluated on a shared, tiered pathway, promising materials will continue to accumulate without the comparative evidence needed to identify which of them deserves to reach a patient. Closing that gap is now the rate-limiting step, and it is one the community can address without waiting for the next coating.

## Figures and Tables

**Table 1:** Antibacterial surface strategies for orthopedic titanium implants, organized by dominant antibacterial mechanism, with representative agents/methods, translational maturity, and the evaluation methods most commonly reported for each.

Strategy	Dominant Mechanism	Representative Agents/Methods	Translational Maturity	Typically Reported Evaluation
Bactericidal topography/nano-structuring	Contact (mechano-bactericidal) + anti-adhesion	Nanopillars/nanosheets via hydrothermal, alkaline or plasma etching (insect-wing inspired)	Strong in vitro; limited in vivo; no load-bearing clinical	SEM of ruptured cells; adhesion/viability counts [11,12]
Antibiotic-loaded/eluting coatings	Release	Gentamicin, vancomycin from resorbable carriers	Clinically translated (fracture fixation) [13]	Elution kinetics; ZOI; CFU after release [14]
Silver/metal-ion/inorganic coatings	Release + contact	Ag (nanoparticle/ion/sputtered film); other inorganic ions preclinically	Ag clinically used (megaprostheses) [13,15]; wider palette preclinical	CFU; ZOI; biofilm assays; ion-release quantification [5,8,14]
Dual osteogenic–antibacterial surfaces	Release (dual agent)	Sr/Ag co-doping; Ag-loaded TiO_2_ nanotubes	Preclinical (mostly in vitro)	Paired antibacterial + osteogenic markers; ion release vs. thresholds [8,16,17]
Plasma-based surface modification	Chemistry + topography (contact/release, design-dependent)	Plasma immersion ion implantation; plasma electrolytic oxidation; magnetron sputtering	Preclinical; durable adhesion may favor translation [18]	Single-strain in vitro screens; adhesion/uniformity [5]
Anti-adhesive/non-fouling coatings	Anti-adhesion (repel attachment)	Zwitterionic polymer brushes (sulfobetaine, carboxybetaine); PEG	Preclinical; mostly non-load-bearing substrates	Protein/bacterial adhesion counts; biofilm assays [19,20]

ZOI, zone of inhibition; CFU, colony-forming units; SEM, scanning electron microscopy.

**Table 2:** Proposed minimum-reporting checklist for antibacterial orthopedic implant surface studies; a starting point toward cross-study comparability rather than a finished standard (Section 7). Items are grouped by evaluation domain, and the final column gives the research stage from which each item is expected: S1, exploratory or discovery-stage work; S2, preclinical translational work in which a defined surface on the final device geometry is advanced as a candidate; S3, clinical evaluation (Section 3.1). Not every item applies to every surface, but each should be reported or explicitly marked not applicable.

Evaluation Domain	Item to Report	Required From
Design	1. Intended antibacterial mechanism(s) declared (release/contact-kill/anti-adhesion)	S1
In vitro	2. Bacterial species, strain origin (reference vs. clinical), and inoculum concentration	S1
In vitro	3. Assay type(s) and endpoint measured (killing, adhesion, or biofilm)	S1
In vitro	4. Incubation time, temperature, and medium	S1
In vitro	5. Quantification metric with units (e.g., log_10_ CFU reduction, % adhesion, biofilm biomass)	S1
Durability	6. Coating adhesion (e.g., ASTM F1044 shear/F1147 tension) and corrosion (ASTM F2129) results	S2
Characterization	7. Direct surface chemical characterization (e.g., XPS, ToF-SIMS) on the final device geometry	S1 (any surface); S2 (final device geometry)
Dual-function	8. Both antibacterial AND osteogenic endpoints, with antibacterial dose interpreted against an explicit cytotoxicity threshold (host/immune response, e.g., macrophage polarization, reported where assessed—emerging, not yet required)	S2 (S1 if a dual-function claim is made)
In vivo	9. Clinically representative, standardized model: species, load-bearing implant, clinically relevant strain, and biofilm/bacterial-load/host-response readouts	S2
Clinical	10. A single, stated infection definition (e.g., MSIS, ICM, or CDC) applied consistently	S2 (in vivo infection outcome); S3 (clinical)

CFU, colony-forming units; XPS, X-ray photoelectron spectroscopy; ToF-SIMS, time-of-flight secondary-ion mass spectrometry; MSIS, Musculoskeletal Infection Society; ICM, International Consensus Meeting; CDC, Centers for Disease Control and Prevention. S1, exploratory/discovery stage; S2, preclinical translational stage; S3, clinical evaluation stage.

**Table 3:** Pilot application of the Table 2 checklist to thirteen open-access primary studies (2022–2025) of antibacterial titanium and titanium-alloy surfaces. Item numbers correspond to Table 2. R, reported; P, partially reported; N, not reported; —, not required at that study’s stage (Section 3.1) or no dual-function claim made. Stage: S1, exploratory; S2, preclinical translational candidate.

Study	Stage	1	2	3	4	5	6	7	8	9	10
Lin et al. 2022 [57]	S2	R	R	R	R	R	P	P	P	R	N
Zhou et al. 2023 [58]	S2	R	P	R	R	R	N	P	P	P	N
Shimabukuro et al. 2024 [59]	S2	R	R	P	R	P	N	P	P	P	N
von Hertzberg-Boelch et al. 2025 [50]	S2	N	P	P	P	P	N	N	P	R	R
Celesti et al. 2022 [60]	S1	R	R	P	R	R	—	P	—	—	—
Rothpan et al. 2023 [61]	S1	R	R	R	R	R	—	N	P	—	—
Fan et al. 2023 [62]	S1	P	R	R	R	R	—	P	P	—	—
Liu et al. 2024 [63]	S1	R	P	R	R	P	—	P	—	—	—
Lallukka et al. 2022 [64]	S1	R	R	P	R	P	—	R	—	—	—
Tani et al. 2024 [65]	S1	P	R	R	R	P	—	R	P	—	—
Wu et al. 2023 [66]	S1	R	N	N	P	N	—	R	P	—	—
Durdu et al. 2024 [67]	S1	N	N	P	P	R	—	P	—	—	—
Parau et al. 2025 [68]	S1	P	N	N	P	P	R	R	—	—	—

Item 6 is not required at stage S1; the Zn-doped CaP study reported it nonetheless. Weighted completeness scores derived from this table are shown in Figure 4B.

## Data Availability

All data generated in this study are contained within the article and its Supplementary Material. The pilot application of the checklist (Table 3, Figure 4) was performed on published open-access articles; the search string, eligibility criteria, the list of scored studies, and the per-item scoring notes are provided as Supplementary Material so that every scoring decision can be independently checked.

## Supplementary Materials

The following supporting information can be downloaded at https://www.mdpi.com/article/10.3390/bioengineering13090999/s1: Supplementary File S1: Pilot application of the Table 2 checklist: search strategy, eligibility, and per-item scoring notes.
