# Supplementary material for: The Standardization and Comparability Gap in Evaluating Antibacterial Surfaces for Load-Bearing Orthopedic Implants: A Critical Review
Source: Bioengineering (Basel). 2026 Aug 27;13(9):999. doi: 10.3390/bioengineering13090999 (PMC13603435; doi:10.3390/bioengineering13090999)
Supplement: Supplementary file 1 [file bioengineering-13-00999-s001.zip › bioengineering-4465052-supplementary.pdf]

Supplementary File S1

Pilot application of the Table 2 checklist: search strategy, eligibility, and per-item scoring notes

S1.1 Purpose

The checklist proposed in Table 2 of the main text is a proposal, and the question a reader is entitled to ask is whether it separates studies whose results can be placed on a common scale from studies whose results cannot. This file documents the retrospective application reported in Section 7, Table 3 and Figure 4 of the main text in enough detail for the exercise to be repeated and for every scoring decision to be checked. The purpose of the exercise was to test the instrument, not to estimate the reporting quality of the field and not to evaluate the scientific quality of the studies scored.

S1.2 Search strategy

Database: PubMed. Search executed 7 August 2026. Search string, verbatim:

```
(antibacterial[Title] OR antimicrobial[Title]) AND (coating[Title] OR surface[Title]) AND
(titanium[Title/Abstract] OR "Ti-6Al-4V"[Title/Abstract]) AND (implant[Title/Abstract] OR
orthopaedic[Title/Abstract] OR orthopedic[Title/Abstract] OR arthroplasty[Title/Abstract]) AND
"free full text"[sb] AND 2022:2026[dp]
```

The search returned 66 records. Records were assessed for eligibility in the order returned by PubMed's relevance ranking, and the first thirteen eligible records were scored, with deliberate coverage of all three mechanism classes (releasing, contact-killing, anti-adhesive). No further records were assessed once thirteen eligible studies had been obtained. The thirteen studies scored were published between 2022 and 2025; the filter's 2026 upper bound reflects the date the search was run.

S1.3 Eligibility criteria

1. Primary study. Reviews, systematic reviews, and meta-analyses were excluded.
2. Substrate is titanium or a titanium alloy.
3. The study evaluates an antibacterial function of the surface of a bone-anchored implant.
4. Full text is open access, so that a reader can verify each scoring decision against the same sentences.
5. Full text is deposited in PubMed Central; records for which only an abstract is available cannot be audited and were excluded.

S1.4 Exclusions applied, with reasons

Two records that met the topical criteria were excluded, and both exclusions are recorded here because they bear on the interpretation of the sample.

- PMC8661698 (Mater Today Bio 2022, nanostructured titanium alloy surface). Only the abstract is deposited in PubMed Central (254 words of body text), so the scoring could not be audited by a reader. Excluded under criterion 5.
- PMC11387750 (Biomater Res 2024, coating on a titanium plate for fracture fixation surgery). The study is specific to trauma fixation, which lies outside the scope declared in Section 3.1 of the main text. Excluded so that the pilot would respect the same scope restriction the review imposes on itself.

S1.5 Studies scored

Reference numbers are those of the main text. Stage was assigned as defined in Section 3.1: S2 where the study includes an in vivo experiment or otherwise advances a defined surface as a translational candidate, and S1 otherwise.

Ref.	Study	Journal	PMCID	Dominant mechanism	Stage
57	Lin et al. 2022	Mater Today Bio	PMC9250043	Release + contact	S2
58	Zhou et al. 2023	Bioact Mater	PMC9127278	Release (Ag + peptide)	S2
59	Shimabukuro et al. 2024	Sci Technol Adv Mater	PMC10854433	Release (Cu)	S2
50	von Hertzberg-Boelch et al. 2025	Front Bioeng Biotechnol	PMC12644090	Release (alkaline)	S2
60	Celesti et al. 2022	Materials	PMC9105612	Anti-adhesion	S1
61	Rothpan et al. 2023	Materials	PMC10649639	Release (TiO2 NP)	S1
62	Fan et al. 2023	J Orthop Surg Res	PMC10680288	Release (MgO)	S1
63	Liu et al. 2024	Small	PMC7616388	Contact (nanoflake)	S1
64	Lallukka et al. 2022	Nanomaterials	PMC9737843	Contact (nisin)	S1
65	Tani et al. 2024	Int J Mol Sci	PMC11394916	Contact (Si3N4)	S1
66	Wu et al. 2023	Front Bioeng Biotechnol	PMC10060813	Release (Cu-Sr)	S1
67	Durdu et al. 2024	Sci Rep	PMC10770057	Contact/anti-adhesion	S1
68	Parau et al. 2025	J Adv Res	PMC12627863	Release (Zn-CaP)	S1

S1.6 Scoring rules

Each of the ten items of Table 2 was scored for each study as follows.

- R (reported) — the information the item asks for is stated explicitly.
- P (partially reported) — the information is present but incomplete or ambiguous. Examples treated as P: a bacterial species named without a collection accession number or other strain designation; an inoculum given as a turbidity standard rather than in colony-forming units; an antibacterial result expressed as a percentage without the denominator or units; surface chemical characterization performed on coupons only rather than on the final implant geometry.
- N (not reported) — the information is absent.
- — (not applicable) — the item is not required at that study's stage under Table 2, or the study makes no dual-function claim, so item 8 does not apply.

Item 7 is stage-conditional: at S1, direct surface chemical characterization of any surface satisfies the item, whereas from S2, it must be performed on the final device geometry. Three exploratory studies therefore score R on item 7 for X-ray photoelectron spectroscopy performed on coupons, while translational candidates that characterized coupons only are scored P. Weighted completeness, plotted in Figure 4B of the main text, is the sum over the items required at that study's stage of 1 for R and 0.5 for P, divided by the number of such items, expressed as a percentage. Because S1 and S2 studies are assessed against different numbers of items, these percentages are comparable within a stage and not across stages, and Figure 4B groups the two stages separately for that reason.

S1.7 Scoring procedure

For each study, the full text was searched for the wording that bears on each item, and the score was assigned from the study's own sentences. Every cell's provisionally scored N was then re-checked with a broader set of search terms before the score was recorded. That second pass corrected four scores: one inoculum was reported as "2.6×10^7

colony-forming units (CFUs)," with the unit spelled out rather than in the abbreviated form searched for first; two inocula appeared in files in which the publisher had inserted zero-width characters between the number and its unit; and one study identified its strains by deletion-mutant genotype rather than by a collection accession number.

S1.8 Per-study scoring notes

Only the determinations that are not self-evident from Table 3 are noted. Item numbers are those of Table 2.

Lin et al. 2022 [57], S2

The highest-scoring study in the sample. Release-killing and contact-killing were evaluated separately, which is the mechanism-matched practice the review advocates; strains were given with ATCC numbers, the inoculum in CFU/mL, and colony counting was used for both mechanisms. Item 6 scored P: a scratch test of coating adhesion was performed but no corrosion testing. Item 7 scored P: XPS was performed on prepared samples rather than on the implant geometry used in vivo. Item 10 scored N: the animal experiment used no stated infection definition.

Zhou et al. 2023 [58], S2

Item 2 scored P: the tested organism is *S. aureus*, for which the collection number ATCC 25922 is given, but the inoculum is reported as 1×10^6 cells/mL counted with a hemocytometer rather than as a viable count in colony-forming units; MRSA appears in the introduction as background rather than as the strain tested. Item 6 scored N: neither coating adhesion nor corrosion was tested, although the study includes a rat implantation experiment. Item 9 scored P: the animal experiment ("*In vivo* bone implantation") evaluates osseointegration and systemic safety in rat femora, and no bacterial challenge was given in vivo, so neither a strain nor a bacterial-load readout is reported for the animal model.

Shimabukuro et al. 2024 [59], S2

Item 2 scored R: the strain is given as MRSA ATCC 43300 and the inoculum as 2.6×10^7 colony-forming units per mL. Items 3 and 5 scored P: viability relative to control rather than an absolute count with units. Item 9 scored P: the in vivo experiment implanted polished discs, so the model is not load-bearing. Item 6 scored N.

von Hertzberg-Boelch et al. 2025 [50], S2

The only study in the sample that combines a load-bearing model with a prospectively stated infection definition, and item 10 is scored R on that basis: a tissue neutrophil-percentage cut-off of 0.7 was defined for the model. Inoculation doses of 10^3, 10^4 and 10^5 CFU are reported, chosen because published infection rates in comparable models differ with dose (40% at 10^3 versus 70% at 10^5 CFU in the earlier series the authors cite); this is the observation cited in Section 3.6 of the main text. Item 1 scored N: no antibacterial mechanism is declared for the coating. Item 7 scored N: no surface chemical characterization of any kind is reported.

Celesti et al. 2022 [60], S1

Anti-adhesive surface, and the assays used are adhesion-based, which is mechanism-matched. Item 3 scored P: adhesion was quantified as CFU per disc, but biofilm was not assessed. Item 8 not applicable: no osteogenic or cytocompatibility endpoint is claimed.

Rothpan et al. 2023 [61], S1

One of two studies that report items 1 to 5 in full. Item 7 scored N: no surface chemical characterization is reported — no XPS, EDS, FTIR, XRD, or equivalent. This determination is the reason item 7 of Table 2 was refined during the pilot, since the original wording could not record the absence of characterization altogether.

Fan et al. 2023 [62], S1

Item 1 scored P: an alkaline mechanism is suggested but not declared as the intended mechanism of the surface.

Liu et al. 2024 [63], S1

A mechanistic study using defined E. coli deletion mutants. Item 2 scored P: strains are specified by genotype (parent, ΔfimA, ΔfliC) rather than by collection accession number, and the inoculum is reported as approximately 10^7 CFU/mL. Item 5 scored P: confocal live/dead imaging without a quantified metric in units. Item 8 not applicable.

Lallukka et al. 2022 [64], S1

Item 3 scored P and item 5 scored P: colonization was assessed without a quantified metric in units. MRSA ATCC 43300 is identified, and the suspension concentration is reported.

Tani et al. 2024 [65], S1

Item 1 scored P: the mechanism of the silicon nitride surface is not declared. Item 5 scored P: biofilm was assessed by crystal-violet absorbance. Item 8 scored P: ALP activity and cell work are reported alongside the antibacterial result, but the antibacterial dose is not interpreted against a stated cytotoxicity threshold.

Wu et al. 2023 [66], S1

The only study in the sample scored R on item 7: EDS element mapping was performed on the 3D-printed implant itself rather than on a coupon. Items 2, 3, and 5 scored N: the organism is named, but no strain designation, inoculum, or quantified antibacterial metric is reported; the antibacterial assessment is presented as fluorescence imaging of phagocytosis.

Durdu et al. 2024 [67], S1

Item 1 scored N, and item 2 scored N: neither the intended mechanism nor a strain designation is given. Item 5 scored R: adhesion is reported as CFU × 10^4/mL with a percentage inhibition.

Parau et al. 2025 [68], S1

The only study scored R on item 6: coating adhesion strength and potentiodynamic corrosion were both measured, although item 6 is not required at stage S1. Item 2 scored N: the organisms are named, but no strain designation is given, and the inoculum is expressed as 0.5 McFarland turbidity rather than in CFU. Items 3 and 5 reflect an antibacterial rate reported as a percentage without an absolute count.

S1.9 Limitations

• The thirteen studies are a convenience sample taken in relevance-ranked order, not a systematic or random sample of the literature, and the percentages reported should not be read as estimates of reporting practice in the field.

• Scoring was performed by the authors and has not been repeated independently. Inter-rater reliability, which a community version of the checklist would require, is untested.

• The score measures what a study reported, not the quality of the science it did. A study may have performed work it did not describe, and a well-reported study is not thereby a good one.

• Restriction to open-access full texts makes the scoring auditable but may not be neutral with respect to reporting practice.
